# Reduced-tillage management enhances soil properties and crop yields in a alfalfa-corn rotation: Case study of the Songnen Plain, China

**DOI:** 10.1038/s41598-019-53602-7

**Published:** 2019-11-19

**Authors:** Jishan Chen, Ruifen Zhu, Qiang Zhang, Xiaolei Kong, Dequan Sun

**Affiliations:** Institute of Pratacultural Science, Heilongjiang Academy of Agricultural Science, Harbin, Heilongjiang 150086 China

**Keywords:** Ecology, Ecosystem ecology

## Abstract

The reduced-tillage (Rt) has been proposed as a strategy to improve soil organic carbon and soil total nitrogen pools. However, little is known of the role of the reduced-tillage compared with the organic (Org) and conventional (Con) management in the Songnen Plain of China. We studied the 4 yr effect of three management strategies (Con, Org and Rt management) on labile soil organic carbon (C) and nitrogen (N) pools, including variation in mineralizable carbon and nitrogen, microbial biomass carbon and nitrogen, dissolved organic carbon and nitrogen in the rotation of alfalfa-corn established in 2009. Soil characteristics including soil organic carbon (SOC), soil total nitrogen (STN), dissolved organic carbon (DOC), dissolved organic nitrogen (DON), microbial biomass carbon (MBC), and microbial biomass nitrogen (MBN) were quantified in samples collected during the 9 yr rotation of 5yr-alfalfa (*Medicago sativa* L.) followed by 4 yr corn (*Zea mays* L.). The mineralizable C was increased in the four years, and although not statistically significant, 12% higher in the fourth year under reduced-tillage than conventional management (268 kg ha^−1^). Soil organic C was increased by 30% under reduced-tillage compared to conventional management (15.5 Mg ha^−1^). Three management strategies showed similar labile N pools in the Con and Org management, but differed in the Rt management. Org management showed significantly lesser mineralizable and inorganic N compared to other strategies, but soil microbial community and comparable crop yield across management strategy in year 4, indicating more efficient N use for organic than other management strategy. In our conditions, reduced-tillage for corn cropping after five years of alfalfa grassland can accumulate labile C and N and improve N utilization to for crop yields in the forage-based rotations. These findings suggest an optimal strategy for using Rt management to enhance soil properties and crop yield in plantation soils and provide a new perspective for understanding the potential role of Rt management in plantation soil.

## Introduction

It is well known that many management practices can improve soil organic carbon (C) and soil total nitrogen (N) pools. Recent studies reported increase in soil organic carbon and soil total nitrogen pools when intensive tillage was changed to reduced- or no-tillage management strategies^1,2^. Strategies to increase soil organic carbon and soil total nitrogen pools may include the use of manure and composts in agro-ecosystem^3^. The effects of different strategies on dynamics of soil organic carbon and nitrogen pools in perennial forage-based rotation, however, is lacking in the Songnen Plain of China.

A typical crop-livestock integrated system in this region of China includes perennial forage crops, mainly alfalfa (*Medicago sativa* L.) or mixtures of alfalfa and perennial grasses for hay, for five to seven years or until its production decline^4,5^. The period of transition from perennial to annual crops in rotation is critical, due to possible loss of soil organic carbon and nutrients accumulated under alfalfa-grass mixtures for several years with no soil disturbance^2^. Alternatively, the use fresh or composted manure in organic forage production system may contribute to maintain or improve soil fertility status by increasing soil organic carbon and nitrogen pool, resulting in improved agroecosystem performance. In organic production system, soil organic carbon and nutrient accumulation are affected by manure or compost application, which also contributes to increasing biomass, activity and diversity of soil microorganisms, as well as to soil microbially mediated soil-building processes^6^. It can also contribute to reduced N loss compared with the conventional management system^7^. But organic production also relies on intensive tillage to cultivate crops due to a lack of alternatives for weed control^8^.

A major challenge in conventional as well as organic management of perennial forage-annual crop production system is loss of soil organic carbon and nutrients due to intensive tillage to establish annual crops in rotation. Reduced tillage management can improve soil quality by conserving soil organic carbon^9,10^, increasing crop residue inputs^11^, augmenting microbial biomass and activity^12^, and increasing root biomass production^2^. Studies show that increase in fine roots and microbial biomass is strongly correlated with increase in soil organic carbon content in agroecosystem^2,13^. The agronomic and soil quality benefits in perennial forage because soil disturbance is dramatically reduced during the conversion process. The benefit of perennial forage-annual crop includes the increase soil organic carbon and nitrogen, but also the possibility of producing equivalent or greater yields compared with the conventional management^14,15^. For example, organic forage-grain crop rotations showed comparable crop yield and higher profit than conventional crop rotations in three long-term forage production strategy in Canada^16^. Similarly, organic and conventional management demonstrated little difference in crop yield in long-term cropping management experiments in Pennsylvania^17^ and no difference in vegetable production in California^18^. Reduced-tillage also increases crop production and profitability by minimizing soil disturbance, improving soil quality, and creating more consistent soil environments for microbial growth and activity^19–22^. Hence, understanding the seasonal and interannual dynamics of labile soil organic C and N pools, including variation in mineralizable C and N, microbial biomass C and N, dissolved organic C and N under various cropping system can help producers design more sustainable cropping management in the Songnen Plain of China.

We evaluated the long-term (4 yr) effect of three management strategies that included organic and reduced-tillage management on soil properties and crop yield compared with conventional management in the Songnen Plain of China. The objective of this study was to (1) quantify the impact of three management strategies on soil organic carbon and nitrogen pools, and crop and forage production, (2) determine which management strategies that enhance soil organic carbon and nitrogen pools, and crop and forage production. It was hypothesized that labile soil organic carbon and nitrogen pools would increase more rapidly under reduced-tillage and organic management system compared with the conventional system. In addition, greater yields of perennial forages and corn grain could be as result of increased in soil organic C and N pools in a generally low fertility soils of the Songnen Plain, China.

## Material and Methods

### Ethics statement

No specific permissions were required for the described field studies and for these locations/activities. The location is not privately owned or protected in any way. The studies did not involve endangered or protected species.

### Experimental site

The field experiment was carried out at the Frigid Forage Research Station located at Lanxi county of Songnen Plain, runned by Heilongjiang Academy of Agricultural Sciences (HASS). The station has an altitude of 160 m, longitude of 125°28′24″E, latitude of 46°32′17″N in Northeast China. The climate is classifed as a typical chillness semiwetness monsoon environment (Table 1). The mean annual precipitation is 469.7 mm, and the mean air temperature is 5.3 °C, with a maximum temperature of 31.2 °C (July) and a minimum temperature of −25.2 °C (January), respectively. The total yearly sunshine duration is 2713 hours and the no frost period is 130 days.

**Table 1:** Monthly total precipitation (mm) and average monthly maximum and minimum temperature (°C) from 2014 to 2017 year.

	2014			2015			2016			2017		
	Tmin (°C)	Tmax (°C)	Rainfall (mm)	Tmin (°C)	Tmax (°C)	Rainfall (mm)	Tmin (°C)	Tmax (°C)	Rainfall (mm)	Tmin (°C)	Tmax (°C)	Rainfall (mm)
Jan.	−31.0	−11.0	4.0	−30.0	−10.0	5.0	−29.0	−10.4	6.0	−32.0	−12.0	3.0
Feb.	−28.0	−8.0	5.0	−27.0	−7.0	7.0	−26.0	−7.4	8.0	−29.0	−9.0	4.0
Mar.	−25.0	−5.0	5.0	−24.0	−4.0	6.0	−23.0	−4.4	7.0	−26.0	−6.0	4.0
Apr.	−18.0	2.0	4.0	−17.0	3.0	6.0	−16.0	2.6	7.0	−19.0	1.0	3.0
May.	0.0	20.0	25.0	1.0	21.0	30.0	2.0	20.6	31.0	−1.0	19.0	24.0
Jun.	6.0	26.0	95.0	7.0	27.0	100.0	8.0	26.6	101.0	5.0	25.0	94.0
Jul.	15.0	35.0	130.0	16.0	36.0	140.0	17.0	35.6	141.0	14.0	34.0	129.0
Aug.	10.0	30.0	120.0	11.0	31.0	120.0	12.0	30.6	121.0	9.0	29.0	119.0
Sep.	6.0	26.0	50.0	7.0	27.0	60.0	8.0	26.6	51.0	5.0	25.0	49.0
Oct.	−5.0	15.0	15.0	−4.0	16.0	20.0	−3.0	15.6	21.0	−6.0	14.0	28.0
Nov.	−20.0	0.0	4.0	−19.0	1.0	5.0	−18.0	0.6	6.0	−21.0	−1.0	3.0
Dec.	−29.0	−9.0	2.0	−28.0	−8.0	2.0	−27.0	−8.4	3.0	−30.0	−10.0	1.0

Notes: Tmin = minimum temperature and Tmax = maximum temperature.

**Figure 1:**
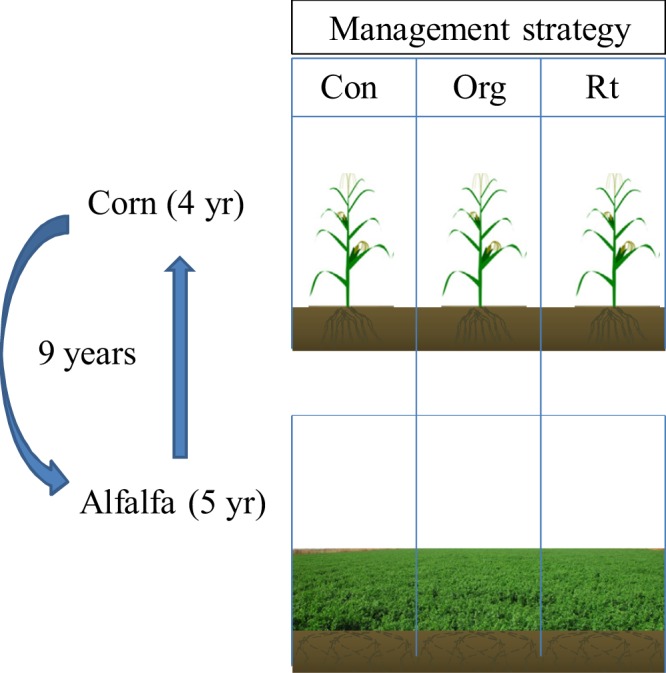
The 9 yr rotation of alfalfa (*Medicago sativa* L.) (5 yr) - corn (*Zea mays* L.) (4 yr) began in 2009. Crop rotations included a homogeneity of alfalfa for the first five years (2009–2013), followed by corn from the sixth year (2014–2017).

The 9 yr rotation of alfalfa (*Medicago sativa* L.) (5 yr) - corn (*Zea mays* L.) (4 yr) began in 2009 with intensively management including the variety, artificial ploughing, weeding and mowing. Beginning of establishing the alfalfa grassland, all seeds were drilled uniformly with a commercial inoculant of *Sinorhizobium*. After seeding, the experiment was reserved to protect the experimental rows from interferential damage. The alfalfa was only used for cutting during the experiment period. Crop rotations included a homogeneity of alfalfa for the first five years (2009–2013), followed by corn from the sixth year (2014–2017) (Fig. 1). The plant density of alfalfa was 1136 × 10^4^ plants ha^−1^ and that for corn was 5.3 × 10^4^ seeds ha^−1^. The alfalfa and corn seed were provided by HASS. These varieties were chosen due to its high adaptability and existing widespread use in the Songnen Plain of China. In this study, the soil is dark loam (mostly Chernozem, FAO Taxonomy) with high melanic humus. The experimental area had an average soil pH of 7.82, an average soil organic matter content of 6.04%, total N content of 0.34%; the contents of NO_3_^–^-N was 4.35%, the contents of NH_4_^+^-N was 6.81% and available P was 22.35 ppm (Olsen method)^23^.

### Experimental design and treatments

In May 2014, 12 plots of 50 × 50 m were established in the rotation of alfalfa-corn. In order to different from the conventional management strategies of annual inorganic fertilization, the randomized complete block design was used. Treatments included (1) organic management (Org), annual organic fertilization with moldboard plowing condition; (2) reduced-tillage management (Rt), Minimum tillage condition with leaving <15% residue cover (Conservation Technology Information Center, CTIC)^24^ and without annual fertilization and (3) conventional management (Con), annual inorganic fertilization with moldboard plowing condition (control). Annual organic and inorganic fertilization were applied in Org and Con, and residue cover applications in Rt. Organic fertilization was applied at 45 t ha^−1^ of cow dung with 3.45 g kg^−1^ N, 0.18 g kg^−1^ P and 1.56 g kg^−1^ K in Org. Inorganic fertilization was fertilized at 337.5 kg ha^−1^ of the compound fertilizer with ratio of 24–7–9 for nitrogen, phosphorus and potassium in Con. Three replicate plots per treatment were established under rainfed condition. During establishing the experimental plots, each plot is 10 meter long and 5 meter wide with inter-row spacing of 60 cm for corn. All seeds were drilled uniformly with three differents management strategies. Soils preparation for conventional and organic management strategy consisted in plowing with a moldboard, disking, and cultivating to a depth of 20 cm (total five tillage passes), with incorporation crop residues into soils, leaving <15% residue cover on the soil surface. The tillage of reduced-tillage management was designed to loosen the surface without inverting the soil^24^ and done with a pass of a Landstar (Kuhn Krause, Inc., Hutchinson, KS), a five-step tillage system that discs, cultivates, and uniformly distributes residues in the seedbed (0–20 cm) and on the soil surface. Before the establishment of the experiment, the entire area was managed under conventionally tilled continuous corn for at least ten years. The soil of the experimental field was not tilled for five years after establishment of the alfalfa grassland at all management strategy.

### Soil properties and microbial biomass determination

Soil from 16 sampling points were collected during spring, early and late summer, and fall seasons at each plot from 2014 to 2017 year. Depth of soil sample collection was 0–30 cm and three layers at soil profiles was sampled, i.e., 0–10 cm, 10–20 cm, and 20–30 cm. Soil core (3.2- × 15-cm) were collected from each transect, composited, thoroughly homogenized, and a ~500 g subsamples were taken to the laboratory. Immediately after the collection samples were kept in a cooler, and stored in a 4 °C refrigerator prior laboratory analyses. All soil were sieved through a 2 mm sieve, and subsamples were stored at −80 °C until further molecular analysis. The others were used to determine the properties of the soil. Analysis of mineralizable C and N, inorganic N, dissolved organic carbon (DOC), dissolved organic nitrogen (DON), microbial biomass carbon (MBC), and microbial biomass nitrogen (MBN) were performed. Soil pH, electrical conductivity (EC), soil organic carbon (SOC), and soil total nitrogen (STN) were determined in samples collected in spring 2014 and fall 2017. The soil texture was determined in soil samples collected in spring 2014. To measure soil bulk density (Db) a separate set of soil samples were collected from sampling points along each transect using a 2.1 cm diameter bulk density probe.

### DNA extraction and bioinformatics analysis

Total DNA was extracted from 0.25 g of soil using the Ezup Column Soil DNA Purification Kit (Sangon Biotech, Beijing, China) according to the manufacturer’s instructions. The 16S rRNA amplicons were sequenced using the Illumina Miseq platform at Novogene (Beijing, China). Briefly, sequence reads were extracted, primers were removed, and the sequences were trimmed to remove low quality sequences. The trimmed and unique sequences were used to define the number of operational taxonomic units (OTUs) at the 97% similarity level. All is to estimate soil microbial community.

### Aboveground biomass and yield measurement

Samples of aboveground biomass of alfalfa-grasses were collected two times in 2009 (July and September) and three times in 2010–2013 (June, August, and September) at the time of hay harvest, which was typically done at 10% flowering of alfalfa. Aboveground biomass samples were collected using a Hege 212 forage plot harvester. Biomass samples were field dried to ~12% moisture, and approximately 500-g subsamples were brought to the laboratory, weighed, oven dried at 70 °C for 48 h, and weighed again to estimate the dry biomass. The sum of aboveground biomass harvested multiple times within a year was considered the annual aboveground biomass. To estimate crop yield every year (2014–2017), corn cobs were allowed dry to ~15% moisture in the field and the total area harvested (0.81 ha per plot) using a John Deer combine harvester, ~100 g subsamples were wet weighed, oven dried at 70 °C for 48 h, and weighed.

### Laboratory analysis methods

The quantification of nitrate (NO_3_^−^) and ammonium (NH_4_^+^) was used to determine soil inorganic N^25^. Soil mineralizable C was analyzed by aerobic incubation for 14 days in a litter glass jar^26^. The incubated samples were used to analyse soil inorganic N and determine mineralizable N. Similarly, DOC, and TDN were determined by the method reported by Ghimire *et al*.^2^. Automatic acidification and the sparging process within the instrument were used to remove the dissolved inorganic C. DON values were calculated by discounting inorganic N from TDN. Fumigation extraction process was used to determine soil MBC and MBN^27^ adopting the efficiency factor 0.41 for both MBC and MBN calculations^28^ without subtracting to non-fumigated control^29^. The hydrometer method was used to determine soil particle-size distribution^30^, electrode for pH and EC by^31^, dry combustion for total C and N (EA1100 Soil C/N analyzer, Carlo Erba Instruments, Milan, Italy), pressure calcimeter for inorganic C^31^, and the gravimetric technique for soil moisture. Total C minus inorganic C was considered the SOC. The core method was used to demine soil bulk density^32^. The V4 hypervariable regions of bacterial 16S rRNA genes were selected for amplification using the primer pair 515 f/806r^33^. PCR conditions and products were as described previously^34^.

### Statistical analysis

Data were conducted using SPSS18.0 for Windows (SPSS Inc., Chicago, Illinois). The split plot in time analysis were used for analyzing soil characteristics such as mineralizable C and N, DOC, DON, MBC, and MBN considering management strategy as a fixed effect, season and year as repeated measurements, and replication as a random term in the model. In this analysis, the year effect was highly significant (*P* < 0.001), but showed a weak relation to effects of management strategy (*P* > 0.05). Thus, all data were re-analyzed for management strategy, season, and management strategy × season interaction within a year considering management strategy as a fixed effect, season as a repeated measure, and replication as a random term in the model (Table 2). In addition, soil microbial community was analyzed by the vegan package in R.

**Table 2:** Analysis of variance result (*P* -values) for labile soil organic carbon and nitrogen pools and other soil properties under conventional (Con), organic (Org) and reduced-tillage (Rt) management strategy.

Parameters	Effects	Year			
		2014	2015	2016	2017
Mineralizable C	Management (M)	0.05	0.01	0.05	0.04
	Season (S)	0.03	<0.01	<0.01	<0.01
	M × S	0.07	0.47	0.58	0.01
Mineralizable N	M	0.48	0.23	0.84	0.08
	S	0.01	<0.01	0.01	0.01
	M × S	0.32	0.09	0.91	<0.01
Inorganic N	M	0.08	0.02	0.99	<0.01
	S	<0.01	0.01	<0.01	<0.01
	M × S	0.29	0.26	0.18	<0.01
DOC	M	0.29	0.22	0.18	0.35
	S	0.05	<0.01	<0.01	<0.01
	M × S	0.27	0.09	0.03	0.52
DON	M	0.29	0.39	0.13	0.48
	S	0.01	<0.01	<0.01	0.28
	M × S	0.38	0.39	0.056	<0.01
MBC	M	0.48	0.02	0.19	0.49
	S	0.01	<0.01	<0.01	<0.01
	M × S	0.39	0.03	0.35	0.03
MBN	M	0.07	0.01	0.02	0.36
	S	0.05	<0.01	<0.01	0.25
	M × S	0.47	0.055	0.49	0.06
**One time measurement**		**Management (M)**		**Year (Y)**	**M** × **Y**
SOC		0.46		0.59	<0.01
STN		0.69		0.39	0.69
pH		0.17		0.16	0.15
EC		0.23		0.51	0.67
Db		0.19		0.15	0.89

Notes: DOC = dissolved organic carbon, DON = dissolved organic nitrogen, MBC = microbial biomass carbon, and MBN = microbial biomass nitrogen, SOC = soil organic carbon, STN = soil total nitrogen, EC = electrical conductivity and Db = bulk density. One time measurement parameters were measured at the beginning (Apr. 2014) and end (Sep. 2017) of the study.

## Results

### Effect of management on the SOC

SOC was affected only by management strategy × year interaction (Table 2). Management strategy showed differences in soil mineralizable C in the four years (Table 3). Rt management showed greater mineralizable C compared to Con and Org management in years 1–4, following a similar trend (Rt > Org > Con) throughout the evaluated period. In the year 2017, mineralizable C in the soil was affected by management strategy × season interaction (Table 2), with greater contents under Rt and lower under Org management compared with Con management in early summer sampling. Soil DOC and MBC contents changed little among management strategy throughout the study, but DOC and MBC contents were significantly increased over the years in all three management strategies (Tables 4–6).

**Table 3:** Labile soil organic carbon under conventional (Con), organic (Org) and reduced-tillage (Rt) management from 2014 to 2017 year.

	Mineralizable C (kg ha^−1^)				DOC (kg ha^−1^)				MBC (kg ha^−1^)			
	2014	2015	2016	2017	2014	2015	2016	2017	2014	2015	2016	2017
Con	181.2 ± 1.12b	210.2 ± 2.13b	220.1 ± 1.14b	230.5 ± 3.17c	161.3 ± 1.06a	160.2 ± 2.14b	160.5 ± 1.02b	170.8 ± 1.33b	460.9 ± 1.42a	410.6 ± 1.12b	400.6 ± 1.19c	455.7 ± 1.88b
Org	180.4 ± 1.08b	200.3 ± 2.16b	240.6 ± 1.15b	270.3 ± 3.16b	160.2 ± 1.02a	200.2 ± 1.12a	180.6 ± 1.82b	280.2 ± 1.67a	500.5 ± 1.85a	720.9 ± 1.77a	880.0 ± 1.33b	890.1 ± 1.57a
Rt	200.6 ± 1.09a	320.5 ± 1.02a	370.8 ± 1.11a	390.9 ± 3.19a	162.1 ± 2.11a	210.9 ± 3.19a	290.5 ± 3.11a	300.8 ± 3.09a	690.9 ± 3.15a	900.7 ± 3.44a	910.9 ± 3.67a	928.6 ± 3.129a

Notes: Means are shown that means with same letter within a year are not significantly different (*P* = 0.05). DOC = dissolved organic carbon and MBC = microbial biomass carbon.

**Table 4:** Nitrogen pools under conventional (Con), organic (Org) and reduced-tillage (Rt) management from 2014 to 2017 year.

	Mineralizable N (kg ha^−1^)				DON (kg ha^−1^)				MBN (kg ha^−1^)				Inorganic N (kg ha^−1^)			
	2014	2015	2016	2017	2014	2015	2016	2017	2014	2015	2016	2017	2014	2015	2016	2017
Con	13.2 ± 0.13a	28.2 ± 0.23a	23.2 ± 0.18a	22.9 ± 0.19c	9.2 ± 0.03a	14.3 ± 0.15a	31.5 ± 0.18a	56.2 ± 0.16a	60.1 ± 0.17a	70.2 ± 0.12b	80.8 ± 0.18b	90.4 ± 0.14a	7.9 ± 0.10a	15.0 ± 0.10a	19.6 ± 0.17a	10.8 ± 0.12c
Org	15.4 ± 0.16a	27.9 ± 0.12a	45.8 ± 0.11a	37.6 ± 0.13b	7.1 ± 0.02a	16.1 ± 0.18a	35.1 ± 0.14a	53.9 ± 0.12a	50.9 ± 0.10a	70.5 ± 0.17b	190.5 ± 0.19b	200.5 ± 0.18a	6.7 ± 0.13a	11.7 ± 0.12a	16.7 ± 0.18a	28.9 ± 0.15b
Rt	18.6 ± 0.17a	34.7 ± 0.23a	47.7 ± 0.16a	60.9 ± 0.11a	10.5 ± 0.03a	19.5 ± 0.17a	46.9 ± 0.10a	65.7 ± 0.18a	80.6 ± 0.16a	110.8 ± 0.15a	280.7 ± 0.15a	280.9 ± 0.15a	5.9 ± 0.16a	20.7 ± 0.14a	22.8 ± 0.12a	40.8 ± 0.19a

Notes: Means are shown that means with same letter within a year are not significantly different (*P* = 0.05). DON = dissolved organic nitrogen and MBN = microbial biomass nitrogen.

**Table 5:** Soil properties under conventional (Con), organic (Org) and reduced-tillage (Rt) management at the beginning (2014 year) and end (2017 year) of the study.

	pH		EC		Db		SOC		TSN	
	2014	2017	2014	2017	2014	2017	2014	2017	2014	2017
			ds m^−1^		Mg m^−3^		Mg ha^−1^		Mg ha^−1^	
Con	7.50	7.52	0.41	0.47	1.50	1.48	19.8^aA^	15.4^bB^	1.70	1.71
Org	7.52	7.09	0.40	0.24	1.54	1.31	21.3^aA^	18.2^bA^	1.78	1.89
Rt	7.51	7.10	0.42	0.30	1.53	1.21	20.7^aA^	25.1^aA^	1.73	2.04

Notes: Means are shown that means with same letter within a year are not significantly different (*P* = 0.05).

EC = electrical conductivity, Db = bulk density, SOC = soil organic carbon, and STN = soil total nitrogen.

**Table 6:** Labile soil organic carbon influenced by management strategy × season interaction in 2017 under conventional (Con), organic (Org) and reduced-tillage (Rt) management.

	Mineralizable C (kg ha^−1^)				MBC (kg ha^−1^)			
	Spring	Early summer	Late summer	Fall	Spring	Early summer	Late summer	Fall
Con	510.7 ± 22.13a	280.5 ± 20.15b	170.6 ± 12.10a	100.8 ± 8.15a	690.8 ± 32.54b	1380.6 ± 42.18b	680.3 ± 26.44b	1030.9 ± 52.66a
Org	560.5 ± 25.17a	160.8 ± 20.17c	160.8 ± 11.16a	120.3 ± 9.16a	790.9 ± 32.21a	1400.8 ± 32.77ab	1000.4 ± 25.19a	1180.6 ± 61.43a
Rt	460.79 ± 26.10a	370.1 ± 21.18a	180.9 ± 13.14a	130.8 ± 7.19a	750.8 ± 30.14ab	1580.9 ± 42.63a	920.8 ± 21.87a	1120.4 ± 53.17a

Notes: Means are shown that means with same letter within a season are not significantly different (*P* = 0.05). MBC = microbial biomass carbon.

### Effect of management on the N pools

Mineralizable N content in the soil was similar among the management strategy in the first three years, but increased as experimental period progress (Table 4). In year 4, however, soil mineralizable N was significantly lesser for Con compared to Org and Rt management. The mineralizable N content in year 4 was affected by management strategy × season interaction (Table 2). Greater soil mineralizable N content was found at Org management compared to Con in the spring season, but the concentration was continuously reduced toward summer and fall seasons in 2017 year (Table 7). STN, pH, EC and bulk density were not affected by management strategy, year or management strategy × year interaction (Table 2). Inorganic N contents in the soil were consistent among the management strategy in year 1, 2 and year 3 (Table 4), but the DON was not affected by management strategy (Table 2). The Org system showed consistently low soil inorganic N throughout the growing seasons, while in the Con and Rt management were sharply increased in early summer and decreased gradually toward late summer and fall (Table 7).

**Table 7:** Nitrogen pools influenced by management strategy × season interaction in 2017 under conventional (Con), organic (Org) and reduced-tillage (Rt) management strategy.

	Mineralizable N (kg ha^−1^)				Inorganic N (kg ha^−1^)			
	Spring	Early summer	late summer	Fall	Spring	Early summer	late summer	Fall
Con	60.5 ± 1.15^a^	90.2 ± 2.34^a^	55.7 ± 2.07^a^	65.6 ± 1.43^a^	16.1 ± 0.55^a^	45.9 ± 2.38^b^	50.1 ± 2.52^b^	35.3 ± 2.25^b^
Org	80.6 ± 1.33^a^	30.5 ± 2.57^b^	20.8 ± 2.05^b^	25.7 ± 1.42^b^	18.7 ± 0.54a	48.4 ± 2.56^b^	30.3 ± 2.51^b^	20.7 ± 2.17^b^
Rt	45.3 ± 1.24^b^	100.7 ± 2.28^a^	40.1 ± 2.37^a^	35.2 ± 1.57^b^	17.8 ± 0.47^a^	90.1 ± 2.44^a^	80.7 ± 2.38^a^	65.5 ± 2.82^a^

Notes: Means are shown that means with same letter within a season are not significantly different (*P* = 0.05).

### Seasonal variations of the SOC and N pools

Seasonal trend of labile SOC components and N pools in year 4 (Tables 2–4), we observed gradual decreases in mineralizable C from summer to fall seasons under reduced-tillage with no soil disturbance for corn cropping and sharper decreases under the tilled treatment. Management strategy showed different seasonal patterns in mineralizable and inorganic N content, with greater values during summer under conventional and reduced-tillage but not for organic management. The DOC and MBC increased, and DON and MBN decreased across all management strategy (Tables 3 and 4). Consistently low soil inorganic N was observed throughout the study with different seasonal trends for management strategy (Table 7), indicating difference in accumulation of inorganic N in soils receiving organic and chemical fertilizers.

### Effect of management on aboveground biomass yield

Aboveground biomass yield was similar across the management strategy in the first year (Table 8), significantly greater under Rt than under Con and Org management in the second and third year. Management strategy resulted in different biomass yield in fourth year. The experiment was carried out for four consecutive years (2014, 2015, 2016 and 2017), where average annual yield from different years was different and variable. In four growing seasons, the best year for aboveground biomass yield was 2017 year followed by 2016 year for Rt management, while the smallest yield was found in 2014 year for Con management.

**Table 8:** Aboveground biomass (from 2014 to 2017 year) of grain yield of corn under conventional (Con), organic (Org) and reduced-tillage (Rt) management.

	Biomass (Mg ha^−1^)			
	2014	2015	2016	2017
Con	10.21 ± 0.13a	10.82 ± 0.10b	10.92 ± 0.18b	10.43 ± 0.15c
Org	10.72 ± 0.16a	10.61 ± 0.11b	10.53 ± 0.11b	11.22 ± 0.12b
Rt	10.91 ± 0.10a	11.22 ± 0.15a	11.77 ± 0.16a	12.32 ± 0.18a

Notes: Means are shown that means with same letter within a year are not significantly different (*P* = 0.05).

### Effect of management on Soil microbial community

**Figure 2:**
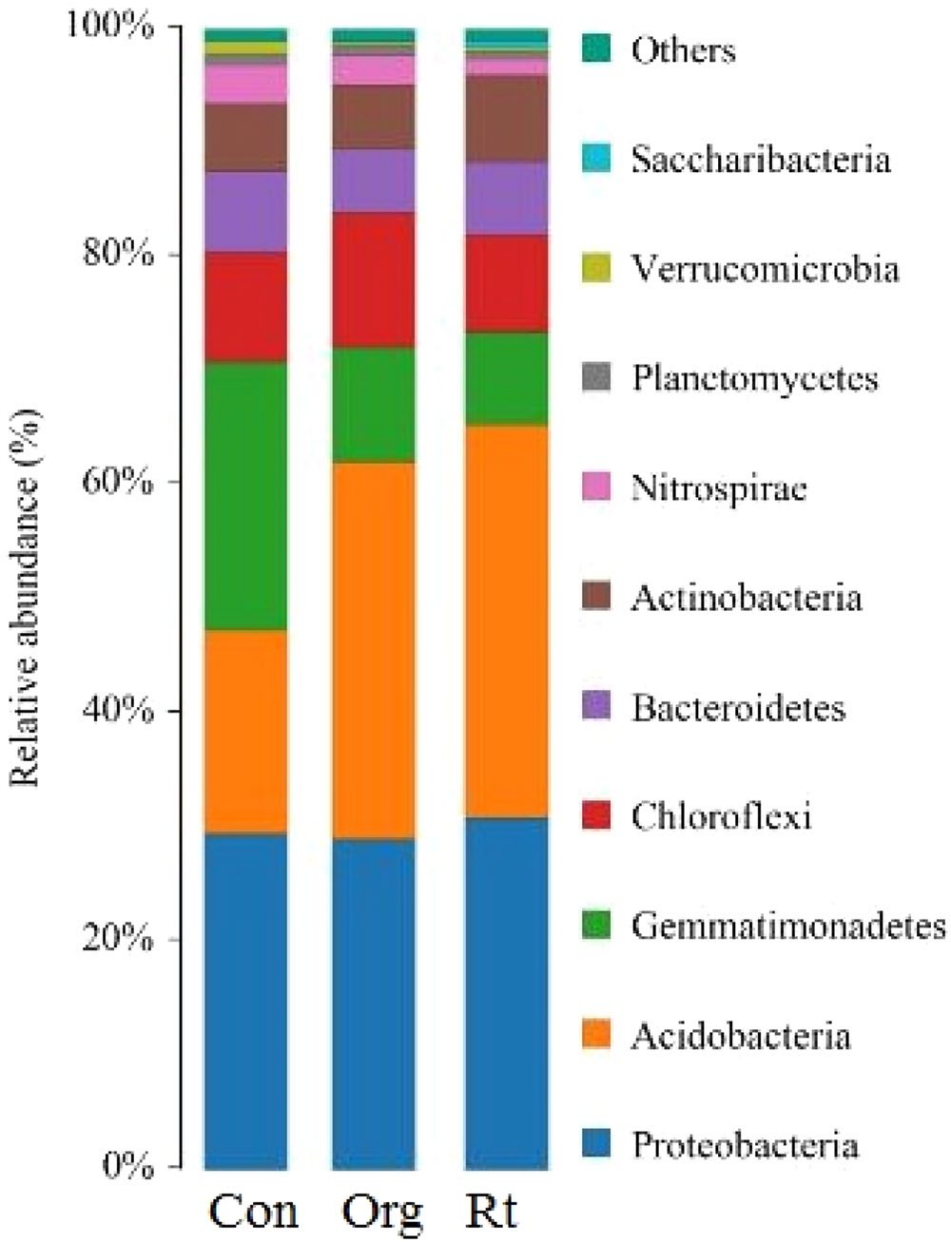
Comparison of the soil bacterial communities at the phylum level in the alfalfa-corn rotation under three management strategies. The relative abundance of the dominant bacterial groups in the soil differed depending on three management strategies. Relative abundance values are based on the proportional frequencies of DNA sequences that could be classified.

More than 26 000 valid reads were obtained for each replicate treatment. The median sequence length of each read was 255 bp. A total of 47 285 OTUs was detected using 97% identity as the cutoff. At the phylum level, the number of Phylum in the Org and Rt management was 22 and 23, which was higher than the number of Phylum in the Con management (Table 9). The dominant phyla were *Proteobacteria* (32.5%), *Acidobacteria* (22.6%), *Gemmatimonadetes* (11.5%), and Chloroflexi (7.6%) (Fig. 2). In the three management strategies, the relative abundance of *Acidobacteria* was lower in the Rt and Org management than in the control (Con), whereas *Gemmatimonadetes* exhibited the opposite trends. The relative abundance of *Proteobacteria* and *Actinobacteria* was higher in the Rt management than in the Org and Con management. In the Con management, apply fertilizer (Urea) increased the relative abundance of *Gemmatimonadetes* but decreased the relative abundance of *Proteobacteria*.

**Table 9:** Soil microbial community under conventional (Con), organic (Org) and reduced-tillage (Rt) management.

	Kindom	Phylum	Class	Order	Family	Genus	Species
Con	1	20	65	100	179	266	189
Org	1	22	74	114	192	291	217
Rt	1	23	75	111	196	294	225

## Discussion

Results support the our hypothesis that Rt would increase soil quality which maintains yields among the three management strategies. In this study, SOC and N pools were higher in forth year under forage-based rotations than in the first year after continuous corn and the highest increase in SOC and N occurred under Rt system, indicating that any reduction in soil disturbance can conserve SOC and N losses. Specifically, soil mineralizable C was higher under Rt management than under Org and Con management in the four years. Because of not tilled during corn cropping in continuous four years after alfalfa, soil mineralizable C under reduced soil disturbance may have contributed to the increase in SOC (Tables 3–5). These results partially support our hypothesis that reduced-tillage and organic management increase SOC and N pools compared with the conventional system. Although not statistically significant, it also showed greater contents of DOC and MBC under Rt than other management strategy (Tables 3 and 4). Some studies have reported that Rt strategy was contribute to SOC accumulation by increasing the amount and diversity of root and soil microbial biomass, crop residue inputs, root exudates, and rhizodeposits^2,19^. Our results here also suggested that Rt strategy was significantly and positively correlated with soil bacterial communities, which was higher the relative abundance of the dominant bacterial groups than in the Con strategy. This conclusion is supported by previous studies^35,36^.

Changes in labile C and N pools in response to alternative management strategy in this study were similar to those reported in previous studies. Previous studies have shown that crop rotations under alternative management strategy may double SOC and N pools^6,37,38^. In the present study, greater DOC, MBC, inorganic N were observed in forth year compared to first year, probably due to management change from monocropping to rotational strategy and reduced soil disturbance, the presence of alfalfa crop in the rotation, and organic manure fertilization to improve soil fertility of the low-fertile soil in the cold, semiarid environment (Table 1). Based on the weather in 2015 and 2016 year, much more precipitation (32.4 and 30.2 mm) were comparative to the average value (28.1 mm) so that more lodging reported by Guo *et al*.^39^ occurred in both years. So both years were conducive to the growth. These weather variations were part of the reasons of annual total yield differences of the same managements, and the interaction (management × year) on aboveground biomass yield. Some studies have reported that increased water saturation will influence soil enzyme activities^40^, mineralization of organic matter and so on^41,42^. In this study, all management strategy showed net gains in SOC and N pools such as mineralizable C and N, MBC, MBN, DOC, and DON during four years (Tables 4 and 5). During the experiment, it showed a benefit in responses of SOC and N pools, and comparable crop yield among management strategy indicates potential for alternative soil fertility strategies to conserve SOC and N and maintain yields.

The similar SOC for Org and Con strategy, indicate possible benefits due to the use of manure in the Org management may take longer than one four-year rotation cycle in perennial forage-based rotations. Long-term studies have shown greater SOC for Org management compared to Con management^10,43^. In the present study, Org management showed greater rates of mineralization and microbial uptake of SOC compared to Con and Rt strategy (Tables 6 and 7). Seasonal trends in mineralizable and inorganic N contents were different for the management strategy, indicating that inorganic fertilizer may have contributes to increased N pools in the Con strategy. The application of nitrogen fertilizers combined with soil disturbance accelerates N mineralization, contributing to increased inorganic N content in the soil^17^. A possible mechanisms responsible for results have reported that Rt system decreased tillage times, reduced the mechanical destruction to soil aggregates, improved the soil physical and chemical properties and increase soil water storage, which can significantly increases crop yields reported by He *et al*.^44,45^.

All management strategy showed an increase in DOC and MBC, and a decrease in DON and MBN, indicating that DOC was mainly root exudates and crop rhizosphere microbial communities, which may have been impacted by growing crops in the rotation. Org system showed consistently low soil inorganic N throughout the study (Tables 4 and 5) and followed different seasonal trends (Tables 6 and 7) compared to other management strategy, indicating differences for organic and chemical fertilizers in soil N accumulation. In fact, soil microbial biomass have been shown to be more sensitive than soil biochemical properties to soil disturbance resulting from intensive tillage practices^46,47^.We noted that the microbial community was also benefited, with greater microbial productivity, saprotrophic bacteria, and gram-positive bacteria and greater efficiency of substrate utilization per unit microbial biomass as reported by Ghimire *et al*.^12^. Management strategy showed differences in SOC and N pools that combined with changes in soil microbiotic properties and root biomass production reported in previous studies^2,13^, suggest an interactions between soil amendment type (chemical vs. organic) and soil tillage strategy.

In Org system in this study, higher N use efficiency is also indicated by comparable yields between Con and Org strategy in all year with different contents of mineralizable and inorganic N (Table 8). This result corroborates with comparable amounts of tissue N content and crop production between Con and Org strategy in a crop rotation study in Iowa^6^ and indicates agronomic and soil quality benefits of Org management compared with Con strategy. However, a longer-term research are needed to explore management strategy with crop rotations and their potential to improve agronomic and soil quality benefits, specifically differences in responses of labile and total C and N under alternative management strategy.

## Conclusion

In the northeast of China, perennial forage-based rotations play an important role for maintaining soil organic matter and crop productivity. SOC and N pools, and crop production were considerably affected by conventional, organic, and reduced-tillage management in this cold, wet, and low fertility environment. Despite consistent increases in labile C and N and comparable yield across management strategy, differences in soil C, N pools and microbial community in experiment year indicate that conventional, organic, and reduced-tillage management impact soil environments differently to mediate crop production. Reduced-tillage and organic strategy can be considered good options to improve agroecosystem performance in highly depleted soils of northeast region, since they produced comparable forage biomass and corn yield to conventional management system in five to six years and increased SOC and N pools. Longer-term monitoring will improve our understanding of relationships among soil C and N pools, seasonal and interannual dynamics, and crop performance under alternative management strategy in perennial forage-based rotations. The effects of the perennial forage-based rotations on fungal community structure and diversity under management strategy need further study.

## Data Availability

The dataset of our study is available from the first author on reasonable request.
